# Research on Kinematic and Static Filtering of the ESKF Based on INS/GNSS/UWB

**DOI:** 10.3390/s23104735

**Published:** 2023-05-14

**Authors:** Zongbin Ren, Songlin Liu, Jun Dai, Yunzhu Lv, Yun Fan

**Affiliations:** 1 Institute of Geospatial Information, Information Engineering University, Zhengzhou 450001, China; rzb13017600350@163.com (Z.R.); daijun502@163.com (J.D.); lvyunzhu11@163.com (Y.L.); 2 School of Aerospace Engineering, Zhengzhou University of Aeronautics, Zhengzhou 450001, China; 3 School of Foreign Studies, Henan Polytechnic University, Jiaozuo 454000, China; fy2635909376@163.com

**Keywords:** UGV, multi-source fusion, error-state Kalman filter (ESKF), kinematic filtering, static filtering

## Abstract

With the widespread development of multiple sensors for UGVs, the multi-source fusion-navigation system, which overcomes the limitations of the use of a single sensor, is becoming increasingly important in the field of autonomous navigation for UGVs. Because federated filtering is not independent between the filter-output quantities, owing to the use of the same state equation in each of the local sensors, a new kinematic and static multi-source fusion-filtering algorithm based on the error-state Kalman filter (ESKF) is proposed in this paper for the positioning-state estimation of UGVs. The algorithm is based on INS/GNSS/UWB multi-source sensors, and the ESKF replaces the traditional Kalman filter in kinematic and static filtering. After constructing the kinematic EKSF based on GNSS/INS and the static ESKF based on UWB/INS, the error-state vector solved by the kinematic ESKF was injected and set to zero. On this basis, the kinematic ESKF filter solution was used as the state vector of the static ESKF for the rest of the static filtering in a sequential form. Finally, the last static ESKF filtering solution was used as the integral filtering solution. Through mathematical simulations and comparative experiments, it is demonstrated that the proposed method converges quickly, and the positioning accuracy of the method was improved by 21.98% and 13.03% compared to the loosely coupled GNSS/INS and the loosely coupled UWB/INS navigation methods, respectively. Furthermore, as shown by the error-variation curves, the main performance of the proposed fusion-filtering method was largely influenced by the accuracy and robustness of the sensors in the kinematic ESKF. Furthermore, the algorithm proposed in this paper demonstrated good generalizability, plug-and-play, and robustness through comparative analysis experiments.

## 1. Introduction

Navigation and positioning are the core technologies of the Internet of Things and location service applications, which occupy a pivotal position in national security and economic construction [1]. With the increasing demand for unmanned, intelligent, and autonomous vehicles in various fields, unmanned ground vehicles (UGVs) with flexibility, low cost, strong adaptability, and other characteristics, are widely used in storage and logistics, search and rescue, detection and excavation, reconnaissance and detonation, and other civilian and military fields [2,3,4]. In outdoor environments, the global navigation satellite system (GNSS) can provide high-accuracy positioning for UGVs [5], but when unmanned vehicles are in urban canyons, or in underground or indoor scenes, the GNSS produces a loss of lock, rejection, and other phenomena, which means that the availability, continuity, and reliability of GNSS signals are not guaranteed.

To address problems such as the inability of single-GNSS positioning to meet production and construction needs, Yang Yuanxi proposed the concept of integrated positioning, navigating, and timing (PNT), the core of which is not to rely excessively on the GNSS and to use all available PNT information sources to implement all-space target positioning, navigation, and timing services [6]. In the indoor environment of GNSS rejection, many scholars have conducted research on different positioning technologies, such as ultra-wide band (UWB) positioning technology [7], the inertial navigation system (INS) [8], wireless local area network (WLAN) positioning technology [9], radio-frequency identification (RFID) positioning technology [10], and Bluetooth positioning technology [11]. Existing communication means, including WLAN, RFID, and Bluetooth, are unable to achieve accurate indoor positioning because of easy disruption; UWB has attracted a great deal of interest because of its high temporal resolution, high reliability, and good obstacle-penetration capabilities [12]. For example, in seamless indoor and outdoor positioning scenes, the authors of [13] conducted experiments related to multi-sensor fusion positioning. Based on the construction of relevant experimental scenes, experiments were conducted using other sensors, such as IMU, GNSS, and UWB, for seamless positioning indoors and outdoors, and the experimental results showed that UWB and IMU can provide high-accuracy positioning for carriers in indoor scenes with GNSS rejection. The authors of [14] proposed a GNSS/INS/UWB tightly coupled, integrated positioning method. In an indoor scene, the method uses UWB-distance measurements to correct INS errors, and in the transition between outdoor and indoor scenes, the method uses a GNSS/INS/UWB tightly coupled, integrated positioning system to ensure the continuity of positioning accuracy and to further improve system reliability. For UWB positioning technology, which offers high overall positioning accuracy, the complexity of indoor environments, building layouts, internal structures, materials, and human movements can cause multipath effects, non-line-of-sight (NLOS) effects, and interference with electronic equipment signals, causing UWB positioning systems to be less accurate or even impossible to locate. An inertial navigation system (INS) cannot be used for indoor positioning alone over a long time period because of the accumulation of errors caused by gyroscope and accelerometer bias and drift.

Single positioning techniques are limited by signal measurements and algorithms in terms of the positioning accuracy that can be achieved [15]. In environments where multiple wireless technologies coexist, fused positioning aims to utilize multiple signal-measurement data or multiple positioning algorithms simultaneously to provide better position estimations [16]. This method is currently one of the main solutions for improving the accuracy and robustness of the integral system. Currently, the distributed Kalman filter and federated Kalman filter (FKF) are used for fusion navigation, with several sensors on the same carrier. The former can incorporate navigation data from multiple sensors by means of parallel data-processing capabilities and strong error correction, and the latter builds on the former by adding the principle of information sharing to improve the accuracy and robustness of the integral filter. Zhang et al. proposed a multi-source information-fusion localization algorithm based on joint Kalman filtering, which is fault-tolerant and reduces computational effort compared to centralized Kalman filtering [17]. The authors of [18] proposed an adaptive federated filtering algorithm, which can calculate the information-distribution coefficient by using prior information and adjust the information-distribution coefficient in real time.

In theory, if the output quantities of each local filter are independent of each other and between the local filter and the output quantity of the main filter, the FKF has integral optimality or near-optimality, as well as high fault tolerance, and is suitable for real-time navigation data. However, in practice, the same equation of state is used between the local filters of the federated filter, resulting in a lack of independence between the main filter and the local sensor outputs and between the local filter outputs, which fails to satisfy the prerequisites of the federated filter and ensures that the fused navigation results are not theoretically rigorous or optimal. By conducting numerical simulations of the three-dimensional state and two-dimensional observation systems, as well as combined velocity and attitude-transfer-alignment-navigation simulation experiments, Yan Gongmin et al. [19] verified that the errors in the suboptimal estimation results of the FKF were excessively large to meet the high-precision-filtering needs of the actual system.

To avoid reusing the carrier equation-of-state information and to solve the problem of the correlation between each local filter and between the local filter and the main filter of the FKF, Yang Yuanxi [20] proposed a kind of sequential kinematic- and static-filtering-fusion navigation, which divides the local filter into kinematic Kalman filtering and static Kalman filtering. After the kinematic Kalman filtering of the output of either observation ephemeris based on the dynamics model with the base sensor or the first sensor, the navigation information of the remaining sensors is added in a sequential form to create a static Kalman filter and, finally, a fusion solution of all the navigation information is obtained. Shi Jianxian et al. [21] proposed a combined GPS/BDS/INS navigation algorithm based on kinematic and static filtering. The algorithm combines the dynamics model of a satellite-navigation system and an inertial navigation system for a kinematic filtering solution, after which the static filter designed by the principle of sequential parity is used to further correct the kinematic filtering results and obtain the final state vector. Wang Yidi et al. [22] proposed a combined pulsar/CNS deep-space-survey navigation method based on improved kinematic and static filtering, which used a UKF in the kinematic filtering to process starlight-angular-distance measurements with a fast sampling rate, strong non-linearity in the measurement equation, and an EKF in the static filtering to process pulsar measurements with a slow sampling rate and obvious linearity in the measurement equation. This avoids the problem of the non-optimality of the fusion filter owing to the use of the same equation of state for each local filter.

In current navigation systems, because of their autonomous characteristics, INSs, which are less susceptible to external interference, are often used to provide continuous, high-update-frequency navigation data. Currently, in most INSs, the error-state Kalman filter (ESKF) is mostly used instead of the traditional Kalman filter. Lu Keke et al. [23] proposed a quadratic attitude-estimation method based on the ESKF, which can avoid covariance matrix singularities and maintain the ability to represent random variable uncertainties. Jun Dai et al. [24] proposed an IMU/GNSS/VO (visual odometry)-based UAV with a robust adaptive-positioning algorithm for use in complex environments, in which a combined ESKF-based navigation model of VO/INS and GNSS/INS was constructed as a local filter of the federated filter to improve the accuracy and robustness of the federated filter in complex environments. The authors of [25] proposed a rigorous attitude-and-position computation algorithm using tightly coupled sensor fusion for multi-antenna, multi-GNSS, and inertial sensor observations, which uses an extended Kalman filter (EKF) and current phase post-processed kinematic (PPK) methods to feed attitude information from multi-antenna GNSS measurements back into the INS, focusing on improving the position- and attitude-measurement accuracy of low-cost UAVs.

In this paper, a kinematic and static filtering method is proposed based on the ESKF and the related properties of kinematic and static filtering, with a UGV as the vehicle. Navigation data from the INS, GNSS, and UWB were used in the filtering. An INS was used as the primary sensor in the filtering, and the GNSS and UWB were used to correct the INS to improve the integral performance of the filtering. In the kinematic ESKF, the ESKF was used instead of the traditional Kalman filter, the error-state vector solved by the kinematic ESKF was injected into the INS, and the velocity and position components of the error-state vector were subsequently zeroed. In the static ESKF, the zeroed error-state vector and its covariance matrix were used as the state vector. Furthermore, the corrected position and velocity errors of both the INS and the UWB were used as the observation equations, which were statically filtered in a sequential form and fed back to the INS to obtain the final fused navigation results. On this basis, simulation and comparison experiments were carried out in this study. In the comparison experiments, the errors of the proposed scheme were compared with the two schemes of the loosely coupled GNSS/INS and the loosely coupled UWB/INS. Through these experiments, the usability of the proposed kinematic and static filtering of the ESKF based on INS/GNSS/UWB was explored. In addition, simulation experiments with different sensor parameters and complex environments were conducted to analyze the generalizability, robustness, and plug-and-play nature of the proposed algorithm. The experimental results indicate that the kinematic and static filtering of the ESKF based on INS/GNSS/UWB proposed in this paper has accuracy advantages over combined navigation algorithms, has good generalizability, and is applicable to different sensors with different accuracies. Its good robustness, the ease with which the filter structure can be changed, and the shielding of contaminated sensors can be applied to seamless indoor and outdoor positioning scenes, providing a filtering basis for seamless indoor- and outdoor-positioning-sensor switching.

This paper is organized as follows. The ESKF algorithm is introduced in Section 2. In Section 3, the kinematic and static filtering of the ESKF based on INS/GNSS/UWB is established. In Section 4, simulation experiments based on the proposed method are reported, and a comparison experiment between the loosely coupled GNSS/INS and the loosely coupled UWB/INS schemes with the same parameter settings is reported, demonstrating the usability of the method proposed in this paper. In Section 5, the generalizability of the proposed algorithm is analyzed by reporting the use of settings with different sensor accuracies. Based on this analysis, the robustness of the filtering was explored by adding the systematic errors of the GNSS and UWB and conducting the simulation experiments in a complex environment. Finally, the conclusions are drawn in Section 6.

## 2. Error-State Kalman Filter

Compared to the Kalman filter, the ESKF can constrain the error state to a position close to the origin, thus avoiding the singularity of the parameters used and ensuring the linearization of the parameters. Because the state and motion vectors of the ESKF are small, the second-order variables of the two vectors can be relatively negligible, thus reducing the error caused by the Taylor expansion in the linearization process. The ESKF defines the true state vector as the sum of the nominal state vector and the error-state vector. The ESKF for inertial navigation systems is divided into four main processes: the nominal-state-prediction process, the error-state-prediction process, the error-state-injection process, and the state-error-nulling process. At moment $t_{k}$, the nominal-state-prediction process and the error-state-prediction process are carried out simultaneously. The prediction is updated for the current moment using the nominal- and error-state quantities from the previous iteration by means of a recursive equation. Next, the true-state estimate at that moment is obtained by injecting the error-state estimate into the nominal-state estimate. Finally, the velocity and position components of the state error are set to zero, and we proceed to the next iteration.

**Figure 1:**
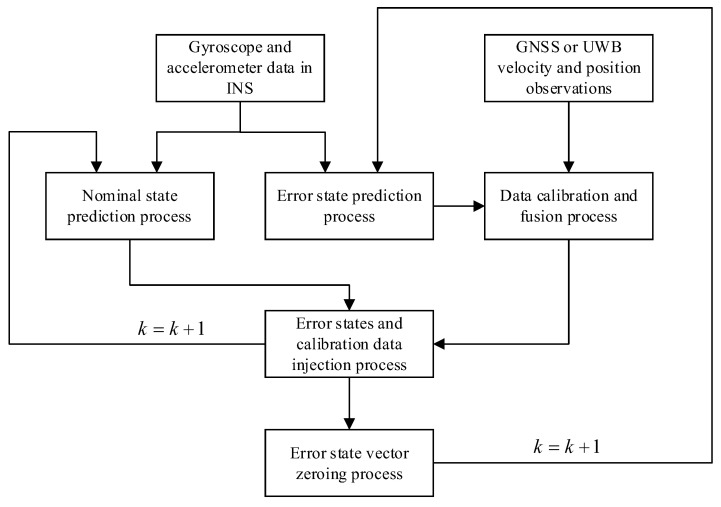
Architecture diagram for the GNSS/INS-based ESKF and UWB/INS-based ESKF.

In this study, two ESKF models for fused navigation were designed in a loosely coupled formulation; these were the GNSS/INS-based ESKF and the UWB/INS-based ESKF. Both fusion models were constructed as ESKF models based on the INS equation of state. The difference in these new models is that after the nominal-state prediction and error-state prediction, the error-state estimates were injected into the calibration-fusion data for the GNSS and UWB. The calibrated-fusion data were then injected into the nominal state estimator, as shown in the structure in Figure 1. Similar to the Kalman filter, the ESKF includes state prediction and measurement prediction, in which the state is updated based on the kinematic model of the INS. Unlike the Kalman filter, the measurement predictions are updated based on the errors in the velocity and position of the GNSS compared to the INS and the errors in the velocity and position of the UWB compared to the INS.

### 2.1. Kinematic Models

In this paper, $X={\left[q,v,p,a_{b},\omega_{b}\right]}^{T}$ is used as the state vector of the overall system, where v is the attitude of the UGV, q is the velocity of the UGV, p is the position of the UGV, $a_{b}$ is the accelerometer bias, and $\omega_{b}$ is the gyroscope bias. In the ESKF, the real-state vector $X_{truth}$ is formed by the nominal-state vector X and the error-state vector δX, and the equation is as follows:

(1) $$X_{truth}=X+\delta X$$

where $X_{truth}={\left[q_{truth},v_{truth},p_{truth},a_{b_{truth}},\omega_{b_{truth}}\right]}^{T}$, $\delta X={\left[\delta q,\delta v,\delta p,\delta a_{b},\delta \omega_{b}\right]}^{T}$, $X={\left[q,v,p,a,\omega \right]}^{T}$.

In the ESKF process, the real-state-dynamics model for the IMU (inertial measurement unit) is modeled in Equation (2):

(2) $$\{\begin{array}{c} {\dot{q}}_{truth}=\frac{1}{2}q_{truth}⊗(\omega_{m}-\omega_{b_{truth}}-\omega_{n})\\ {\dot{v}}_{truth}=C_{b\;truth}^{n}(a_{m}-a_{b_{truth}}-a_{n})\\ {\dot{p}}_{truth}=v_{truth}\\ {\dot{a}}_{b_{truth}}=a_{\omega }\\ {\dot{\omega }}_{b_{truth}}=\omega_{\omega } \end{array}$$

where $a_{n}$ and $\omega_{n}$ represent acceleration and angular velocity measurements and white noise, respectively. The $a_{\omega }$ and $\omega_{\omega }$ represent acceleration bias and angular velocity bias, respectively. $C_{b\;truth}^{n}$ represents the rotation matrix of the real state, which in the INS system is the coordinate-transformation matrix from the carrier coordinate system b to the navigation coordinate system n. ⊗ represents the quaternion multiplication operation and the extraction of the vector part of the result. The nominal-state IMU-dynamics model is presented as Equation (3):

(3) $$\{\begin{array}{c} \dot{q}=\frac{1}{2}q⊗(\omega_{m}-\omega_{b})\\ \dot{v}=C_{b}^{n}(a_{m}-a_{b})\\ \dot{p}=v\\ {\dot{a}}_{b}=0\\ {\dot{\omega }}_{b}=0 \end{array}$$

where $C_{b}^{n}$ represents the rotation matrix of the nominal state. The error-state dynamics model of the IMU is obtained from the above equation, as shown in Equation (4):

(4) $$\{\begin{array}{c} \delta \dot{\theta }={\left[\omega_{m}-\omega_{b}\right]}_{\times }\delta \theta -\delta \omega_{b}-\omega_{n}\\ \delta \dot{v}=-C_{b}^{n}{\left[a_{m}-a_{b}\right]}_{\times }\delta \theta -C_{b}^{n}\delta a_{b}-Ra_{n}\\ \delta \dot{p}=\delta v\\ \delta {\dot{a}}_{b}=a_{\omega }\\ \delta {\dot{\omega }}_{b}=\omega_{\omega } \end{array}$$

where ${\left[a\right]}_{\times }$ represents the antisymmetric matrix of vector a.

Since Equations (2)–(4) are all continuous-form equations, they need to be discretized. After discretization, the recursive equation for the nominal-state dynamics model of the IMU at moment $t_{k}$ is obtained, as shown in Equation (5):

(5) $$\{\begin{array}{c} q_{k+1}=q_{k}⊗q_{k}[(\omega_{mk}-\omega_{bk})\Delta t]\\ v_{k+1}=v_{k}+C_{bk}^{n}[a_{mk}-a_{bk}]\Delta t\\ p_{k+1}=p_{k}+v_{k}\Delta t+\frac{1}{2}[C_{bk}^{n}(a_{mk}-a_{bk})]\Delta t^{2}\\ a_{b(k+1)}=a_{bk}\\ \omega_{b(k+1)}=\omega_{bk} \end{array}$$

where Δt represents the sampling interval of the IMU. The error-state dynamics model of the IMU at moment $t_{k}$ is derived as shown in Equation (6):

(6) $$\{\begin{array}{c} \delta \theta_{k+1}=C_{bk}^{nT}[(\omega_{mk}-\omega_{bk})\Delta t]\delta \theta_{k}-\delta \omega_{bk}\Delta t-w_{\theta k}\\ \delta v_{k+1}=\delta v_{k}-C_{bk}^{n}{\left[a_{mk}-a_{bk}\right]}_{\times }\delta \theta \Delta t-R_{k}\delta a_{bk}\Delta t-w_{vk}\\ \delta p_{k+1}=\delta p_{k}+\delta v_{k}\Delta t\\ \delta a_{b(k+1)}=\delta a_{bk}+w_{ak}\\ \delta \omega_{b(k+1)}=\delta \omega_{bk}+w_{\omega k} \end{array}$$

where $w_{\theta k}$ represents the Gaussian random pulse noise at attitude. The $w_{vk}$ represents the Gaussian random pulse noise at velocity. The $w_{ak}$ represents the Gaussian random pulse noise at acceleration bias. The $w_{\omega k}$ represents the Gaussian random pulse noise at angular velocity bias. Their covariance is defined by Equation (7):

(7) $$\{\begin{array}{c} w_{\theta }=\sigma_{a_{n}}^{2}\Delta t^{2}I\\ w_{v}=\sigma_{\omega_{n}}^{2}\Delta t^{2}I\\ w_{a}=\sigma_{a_{\omega }}^{2}\Delta t^{2}I\\ w_{\omega }=\sigma_{\omega_{\omega }}^{2}\Delta t^{2}I \end{array}$$

where $\sigma_{a_{n}}^{2}$ and $\sigma_{\omega_{n}}^{2}$ represent the standard deviation of Gaussian white noise for acceleration and angular velocity, respectively. The $\sigma_{a_{\omega }}^{2}$ and $\sigma_{\omega_{\omega }}^{2}$ represent the standard deviation of Gaussian white noise for acceleration bias and angular velocity bias. The I represents the unit matrix of 3 × 3.

### 2.2. ESKF State-Prediction Model

Under discrete conditions, the nominal-state vector can be defined as $x_{k}={\left[q_{k},v_{k},p_{k},a_{k},\omega_{k}\right]}^{T}$, the error-state vector as $\delta x_{k}={\left[\delta q_{k},\delta v_{k},\delta p_{k},\delta a_{bk},\delta \omega_{bk}\right]}^{T}$, the IMU acceleration and angular velocity noise-measurement vector as $u_{mk}={\left[a_{mk},\omega_{mk}\right]}^{T}$, and the IMU attitude, velocity, and acceleration zero-bias and angular velocity zero-bias noise vectors as $w_{k}={\left[w_{\theta k},w_{vk},w_{ak},w_{\omega k}\right]}^{T}$. Bringing the vectors defined above into Equation (5), the recursive formula for the nominal-state vector at moment a is denoted by Equation (8):

(8) $$x_{k+1}=f(x_{k},u_{mk})$$

where f(⋅) represents the recursive function of the nominal-state vector. Combining Equation (6) and linearizing it according to Taylor’s formula, the recursive formula for the error-state vector at moment $t_{k+1}$ is derived as follows:

(9) $$\begin{array}{cc} \delta x_{k+1} & =f_{\delta }(x_{k},\delta x_{k},u_{mk},w_{k})\\ & =(I+F_{x_{k}}\Delta t)\delta x_{k}+G_{w_{k}}\Delta tw_{k}\\ & =\Phi_{k+1,k}\delta x_{k}+\Gamma_{k+1,k}w_{k} \end{array}$$

where $f_{\delta }(\cdot )$ represents the recursive function of the error-state vector. The $F_{x_{k}}$ and $G_{w_{k}}$ represent the Jacobi matrices corresponding to the error-state vector and the noise-state vector, respectively. The $\Phi_{k+1,k}$ is the state-transfer matrix from moment $t_{k}$ to moment $t_{k+1}$.

The $\Gamma_{k+1,k}$ is the noise-transfer matrix from moment $t_{k}$ to moment $t_{k+1}$. The specific expressions of $F_{x_{k}}$ and $G_{w_{k}}$ are as shown in Equation (10).

(10) $$\begin{array}{c} F_{x_{k}}=\left[\begin{array}{ccccc} C_{b}^{n}{\left[\omega_{mk}-\omega_{bk}\right]}_{\times } & 0_{3\times 3} & 0_{3\times 3} & 0_{3\times 3} & -I_{3\times 3}\\ -C_{b}^{n}{\left[a_{mk}-a_{bk}\right]}_{\times } & I_{3\times 3} & -C_{b}^{n} & 0_{3\times 3} & 0_{3\times 3}\\ 0_{3\times 3} & I_{3\times 3} & I_{3\times 3} & 0_{3\times 3} & 0_{3\times 3}\\ 0_{3\times 3} & 0_{3\times 3} & 0_{3\times 3} & I_{3\times 3} & 0_{3\times 3}\\ 0_{3\times 3} & 0_{3\times 3} & 0_{3\times 3} & 0_{3\times 3} & I_{3\times 3} \end{array}\right],\\ G_{w_{k}}=\left[\begin{array}{cccc} -I_{3\times 3} & 0_{3\times 3} & 0_{3\times 3} & 0_{3\times 3}\\ 0_{3\times 3} & -I_{3\times 3} & 0_{3\times 3} & 0_{3\times 3}\\ 0_{3\times 3} & 0_{3\times 3} & 0_{3\times 3} & 0_{3\times 3}\\ 0_{3\times 3} & 0_{3\times 3} & I_{3\times 3} & 0_{3\times 3}\\ 0_{3\times 3} & 0_{3\times 3} & 0_{3\times 3} & I_{3\times 3} \end{array}\right] \end{array}$$

According to Equation (9), the covariance matrix $\Sigma_{x_{k+1}}$ of error-state vector $\delta x_{k+1}$ at moment $t_{k+1}$ is derived as shown in Equation (11):

(11) $$\Sigma_{x_{k+1}}=\Phi_{k+1,k}\Sigma_{x_{k}}\Phi_{k+1,k}^{T}+\Gamma_{k+1,k}Q_{w}\Gamma_{k+1,k}^{T}$$

where $\Sigma_{x_{k}}$ represents the covariance matrix of error-state vector $\delta x_{k}$ at moment $t_{k}$. The $Q_{w}$ represents the state-noise matrix, which is represented as follows:

(12) $$Q_{w}=\left[\begin{array}{ccccc} w_{\theta k} & 0_{3\times 3} & 0_{3\times 3} & 0_{3\times 3} & 0_{3\times 3}\\ 0_{3\times 3} & w_{vk} & 0_{3\times 3} & 0_{3\times 3} & 0_{3\times 3}\\ 0_{3\times 3} & 0_{3\times 3} & 0_{3\times 3} & 0_{3\times 3} & 0_{3\times 3}\\ 0_{3\times 3} & 0_{3\times 3} & 0_{3\times 3} & w_{ak} & 0_{3\times 3}\\ 0_{3\times 3} & 0_{3\times 3} & 0_{3\times 3} & 0_{3\times 3} & w_{\omega k} \end{array}\right]$$

### 2.3. ESKF Measurement-Prediction Model

The accelerometer and gyroscope possess bias errors, causing the IMU to drift during the integration of attitude estimation, which gradually increases over time. In this paper, the velocity and position information obtained by the GNSS and UWB are used to correct the IMU observations, thus correcting the error-state estimates of the ESKF to reduce the errors due to accelerometer and gyroscope bias during the fusion of the overall filter process. The GNSS/INS-based ESKF observation model is defined as shown in Equation (13):

(13) $$L_{k}^{GNSS}=\left[\begin{array}{c} v_{INS}-v_{GNSS}\\ p_{INS}-p_{GNSS} \end{array}\right]=H_{k}^{GNSS}x_{k}+v_{k}^{GNSS}$$

where $L_{k}^{GNSS}$ represents the observation vector of the GNSS/INS-based ESKF. The $v_{INS}$ and $v_{GNSS}$ represent velocity measurements obtained from the INS and GNSS, respectively. The $p_{INS}$ and $p_{GNSS}$ represent position measurements obtained from the INS and GNSS, respectively. The $H_{k}^{GNSS}=\left[\begin{array}{ccccc} 0_{3\times 3} & I_{3\times 3} & 0_{3\times 3} & 0_{3\times 3} & 0_{3\times 3}\\ 0_{3\times 3} & 0_{3\times 3} & I_{3\times 3} & 0_{3\times 3} & 0_{3\times 3} \end{array}\right]$ represents the observation-design matrix of the GNSS/INS-based ESKF. The $v_{k}^{GNSS}={\left[\begin{array}{cc} n_{v}^{GNSS} & n_{p}^{GNSS} \end{array}\right]}^{T}$ represents the random-measurement-noise matrix of the GNSS/INS-based ESKF. The $n_{v}^{GNSS}$ and $n_{p}^{GNSS}$ are velocity-observation noise and position-observation noise, respectively, both of which are Gaussian white noise.

Furthermore, the observation model for the UWB/INS-based ESKF is defined as shown in Equation (14):

(14) $$L_{k}^{UWB}=\left[\begin{array}{c} v_{INS}-v_{UWB}\\ p_{INS}-p_{UWB} \end{array}\right]=H_{k}^{UWB}x_{k}+v_{k}^{UWB}$$

where $L_{k}^{UWB}$ represents the observation vector of the UWB/INS-based ESKF. The $v_{UWB}$ represents velocity measurements obtained from the UWB. The $p_{UWB}$ represents position measurements obtained from the UWB. The $H_{k}^{UWB}=\left[\begin{array}{ccccc} 0_{3\times 3} & I_{3\times 3} & 0_{3\times 3} & 0_{3\times 3} & 0_{3\times 3}\\ 0_{3\times 3} & 0_{3\times 3} & I_{3\times 3} & 0_{3\times 3} & 0_{3\times 3} \end{array}\right]$ represents the observation-design matrix of the UWB/INS-based ESKF. The $v_{k}^{UWB}={\left[\begin{array}{cc} n_{v}^{UWB} & n_{p}^{UWB} \end{array}\right]}^{T}$ represents the random-measurement-noise matrix of the UWB/INS-based ESKF. The $n_{v}^{UWB}$ and $n_{p}^{UWB}$ are velocity-observation noise and position-observation noise, respectively, both of which are Gaussian white noise.

According to the basic principle of the Kalman filter, taking the ESKF based on the GNSS/INS as an example, the updated equation of the observation model at moment $t_{k+1}$ is obtained as shown in Equation (15):

(15) $$\left\{ \begin{array}{c} V_{k+1}^{GNSS}=H_{k}^{GNSS}\delta x_{k+1}-L_{k+1}^{GNSS}\\ \Sigma_{V_{k+1}^{GNSS}}=H_{k}^{GNSS}\Sigma_{x_{k+1}}H_{k}^{GNSS}{}^{T}+\Sigma_{k+1}\\ K_{k+1}^{GNSS}=\Phi_{k+1,k}H_{k}^{GNSS}\Sigma_{V_{k+1}^{GNSS}}^{-1}\\ \delta {\hat{x}}_{k+1}=\delta x_{k+1}-K_{k+1}^{GNSS}V_{k+1}^{GNSS}\\ \Sigma_{\delta {\hat{x}}_{k+1}}=(I-K_{k+1}^{GNSS}H_{k}^{GNSS})\Sigma_{{\bar{X}}_{k-1}}(I-H_{k}^{GNSS}{}^{T}K_{k+1}^{GNSS}{}^{T})+K_{k+1}^{GNSS}\Sigma_{k+1}K_{k+1}^{GNSS}{}^{T} \end{array}\right.$$

where $V_{k+1}^{GNSS}$ represents the innovation vector of the GNSS/INS-based ESFK. The $\Sigma_{V_{k+1}^{GNSS}}$ represents the covariance matrix of the innovation vector. The $K_{k+1}^{GNSS}$ represents the Kalman Gain. The $\delta {\hat{x}}_{k+1}$ represents the estimated error-state vector at moment $t_{k+1}$. The $\Sigma_{\delta {\hat{x}}_{k+1}}$ represents the covariance matrix of $\delta {\hat{x}}_{k+1}$. Similarly, the updated equation of the observation model of UWB/INS-based ESKF at moment $t_{k+1}$ is obtained.

### 2.4. ESKF Error-State-Vector Injection and Zeroing

According to Equations (1), (9), and (15), the formula for injecting the error-state vector into the nominal-state vector at moment a to obtain the true-state vector is derived as shown in Equation (16):

(16) $${\hat{x}}_{k+1}=x_{k+1}+\delta {\hat{x}}_{k+1}$$

where ${\hat{x}}_{k+1}$ represents the estimated truth-state vector at moment $t_{k+1}$. The $x_{k+1}$ represents the estimated nominal-state vector at moment $t_{k+1}$. Based on Equation (16), the components are represented in Equation (17).

(17) $$\{\begin{array}{c} {\hat{q}}_{k+1}=q_{k+1}⊗q\delta {\hat{\theta }}_{k+1}\\ {\hat{v}}_{k+1}=v_{k+1}+\delta {\hat{v}}_{k+1}\\ {\hat{p}}_{k+1}=v_{k+1}+\delta {\hat{p}}_{k+1}\\ {\hat{a}}_{b_{k+1}}=a_{b_{k+1}}+\delta {\hat{a}}_{b_{k+1}}\\ {\hat{\omega }}_{b_{k+1}}=\omega_{b_{k+1}}+\delta {\hat{\omega }}_{b_{k+1}} \end{array}$$

To reduce error accumulation, the velocity and position components of the error-state vector are zeroed, as shown in Equation (18):

(18) $$\delta {\hat{x}}_{k+1}^{0}=\delta {\hat{x}}_{k+1}+G\delta {\hat{x}}_{k+1}$$

where $G=\left[\begin{array}{ccc} 0_{3\times 3} & -I_{6\times 6} & 0_{6\times 6} \end{array}\right]$ represents the zeroing matrix.

## 3. Kinematic and Static Filtering of the ESKF Based on INS/GNSS/UWB

**Figure 2:**
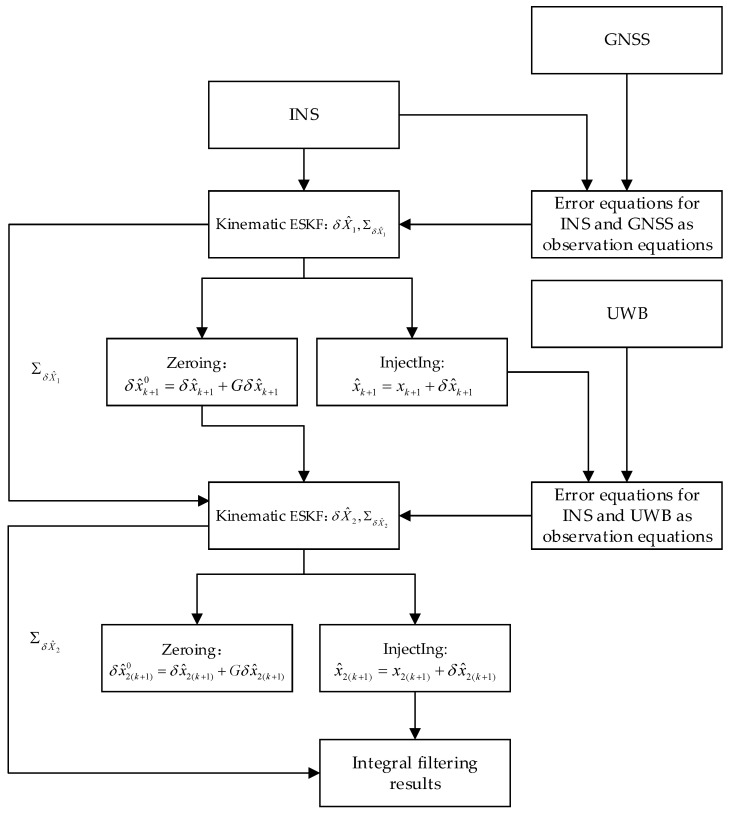
Kinematic and static filtering of the ESKF based on INS/GNSS/UWB structure diagram.

The entire structure of the ESKF-based kinematic and static filter is shown in Figure 2. The integral filter is mainly divided into the kinematic ESKF and static ESKF. First, the IMU navigation data in the INS are used as the model of the kinematic ESKF. The position and velocity errors of the INS and GNSS were used as the observation equations of the kinematic ESKF. Subsequently, the kinematic ESKF solution is calculated to obtain the filtered solution of the kinematic ESKF with its covariance. Since the kinematic ESKF filter results in an error-state vector, the kinematic ESKF filter solution needs to be injected into the nominal-state vector. In addition, the velocity and position components of the error-state vector of the kinematic ESKF need to be set to zero. On this basis, the zeroed error-state vector and the covariance of the kinematic ESKF’s estimated state quantity are passed to the static ESKF in a sequential form as the initial value of the static ESKF. The position and velocity errors of the INS and GNSS are used as the observation equations of the static ESKF, and the static ESKF is solved. The error-state vector of the filtered solution of the static ESKF is then injected into the nominal-state vector of the INS to obtain the true-state vector of the INS after the static ESKF after zeroing the velocity and position components of the error-state vector. Finally, the truth-state quantities obtained from the last static ESKF are output as a result of the navigation of the entire system.

### 3.1. Kinematic ESKF Process

Since different sensors on the same carrier theoretically have the same state vector, the state equations among the different subsystems all coincide with the main system’s state equations. According to Equations (9) and (15), the GNSS/INS-based kinematic ESKF filter solution at moment $t_{k+1}$ can be obtained as shown in Equation (19):

(19) $$\delta {\hat{x}}_{k+1}=\delta x_{k+1}-K_{k+1}^{GNSS}V_{k+1}^{GNSS}$$

where $K_{k+1}^{GNSS}$ represents the Kalman gain of the kinematic ESKF, as represented by Equation (20).

(20) $$K_{k+1}^{GNSS}=\Phi_{k+1,k}H_{k}^{GNSS}{\left(H_{k}^{GNSS}\Sigma_{x_{k+1}}H_{k}^{GNSS}{}^{T}+\Sigma_{k+1}\right)}^{-1}$$

where $\Sigma_{\delta {\hat{x}}_{k+1}}$ represents the covariance matrix of $\delta {\hat{x}}_{k+1}$, as shown in Equation (21):

(21) $$\Sigma_{\delta {\hat{x}}_{k+1}}=(I-K_{k+1}^{GNSS}H_{k}^{GNSS})\Sigma_{{\bar{X}}_{k-1}}(I-H_{k}^{GNSS}{}^{T}K_{k+1}^{GNSS}{}^{T})+K_{k+1}^{GNSS}\Sigma_{k+1}K_{k+1}^{GNSS}{}^{T}$$

According to Equations (16) and (18), when the error-state vector $\delta {\hat{x}}_{k+1}$ is injected into the nominal state vector $x_{k+1}$ of the INS, the INS truth-state vector ${\hat{x}}_{k+1}$ and the error-state vector $\delta {\hat{x}}_{k+1}^{0}$ after setting to zero are obtained.

### 3.2. Static ESKF Process

In the static ESKF, to avoid reusing the kinetic model forecasts of the state vector and its covariance matrix at moment $t_{k}$, the kinematic ESKF filter solution is used to obtain the error-state vector $\delta {\hat{x}}_{k+1}^{0}$ and the covariance matrix $\Sigma_{{\hat{X}}_{1k}}$ of the error-state vector after zeroing as the static ESKF state vector $x_{k+1}$ and its covariance matrix $\Sigma_{x_{k+1}}$. At this point, the state equation of the static ESKF is shown in Equation (22).

(22) $$\begin{array}{l} \delta x_{k+1}=\delta {\hat{x}}_{1(k+1)}^{0}\\ \Sigma_{x_{k+1}}=\Sigma_{{\hat{x}}_{1(k+1)}} \end{array}$$

The error between the truth-state vector ${\hat{x}}_{1(k+1)}$ from the kinematic ESKF filter solution and the position and velocity data from the UWB observation is used as the observation equation. Equations (9), (14) and (15) are used to obtain the static ESKF filter solution based on the UWB/INS, as shown in Equation (23):

(23) $$\delta {\hat{x}}_{2(k+1)}=\delta x_{2(k+1)}-K_{2(k+1)}^{UWB}V_{2(k+1)}^{UWB}$$

where $K_{2(k+1)}^{UWB}$ represents the Kalman gain of the static ESKF, as shown in Equation (24).

(24) $$K_{2(k+1)}^{UWB}=\Phi_{k+1,k}H_{2k}^{UWB}{\left(H_{2k}^{UWB}\Sigma_{{\hat{x}}_{1(k+1)}}H_{k}^{UWB}{}^{T}+\Sigma_{2(k+1)}\right)}^{-1}$$

where $\Sigma_{\delta {\hat{x}}_{2(k+1)}}$ represents the covariance matrix of $\delta {\hat{x}}_{2(k+1)}$, as shown in Equation (25).

(25) $$\Sigma_{\delta {\hat{x}}_{2(k+1)}}=(I-K_{2(k+1)}^{UWB}H_{k}^{UWB})\Sigma_{{\hat{x}}_{1(k+1)}}(I-H_{k}^{UWB}{}^{T}K_{2(k+1)}^{UWB}{}^{T})+K_{2(k+1)}^{UWB}\Sigma_{2(k+1)}K_{2(k+1)}^{UWB}{}^{T}$$

Finally, by injecting the error-state vector $\delta {\hat{x}}_{2(k+1)}$ into the nominal-state vector $x_{2(k+1)}$, the actual state vector ${\hat{x}}_{2(k+1)}$ is obtained. The ${\hat{x}}_{2(k+1)}$ is output as a navigation solution for the integral system. In addition, the error-state vector $\delta {\hat{x}}_{2(k+1)}$ of the static ESKF needs to be zeroed, according to Equation (18).

## 4. Simulation-Experiment Verification

The following simulation experiments were developed and implemented based on the PSINS toolbox by Prof. Yan Gongmin of the Northwestern Polytechnic University.

### 4.1. Coordinate- and Trajectory-Simulation Settings

**Figure 3:**
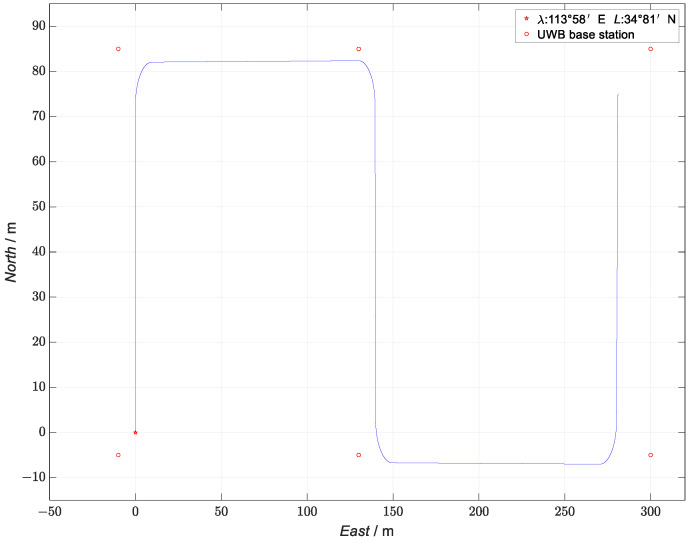
True-trajectory simulation.

As the coordinate systems used by the INS, GNSS, and UWB are distinct, we converted the angular velocity and acceleration data measured by the IMU with respect to the geocentric inertial coordinate system and the xyz coordinate data measured by the UWB with respect to the local coordinate system into the coordinate data of the navigation-coordinate system (n system), which is denoted by $o_{n}x_{n}y_{n}z_{n}$. As the navigation -coordinate system was the reference coordinate system, the “East–North–Sky” geographic coordinate system (*g* system) was selected as the navigation reference coordinate system, represented by $o_{g}x_{g}y_{g}z_{g}$, where the origin was defined as the center of gravity of the UGV, the $o_{g}x_{g}$ axis denoted the geographic east, the $o_{g}y_{g}$ axis denoted the geographic north, and the $o_{g}z_{g}$ axis denoted the sky perpendicular to the local rotating ellipsoid, and the three axes formed a right-handed coordinate system. In addition, the coordinates of the UGV under the g system were expressed in terms of longitude λ, latitude L, and ellipsoidal altitude h. The DW3000 was used as the base station for the UWB simulation and received the signals from the tags mounted on the carrier. The tag information received by the base station was used to locate the tag using the TDOA (time difference of arrival) algorithm. The specific location of the base station is shown in Figure 3, with the red circle representing the UWB base station.

To verify the performance of the proposed INS/GNSS/UWB-based ESKF kinematic and static filter algorithm, relevant simulation experiments were carried out. The initial position of the UGV (represented as a star point on the diagram) was $113^{\circ }58^{\prime }\;E$, $34^{\circ }81^{\prime }\;N$, the initial attitude (yaw, roll and pitch) was $\left[0^{\circ };0^{\circ };0^{\circ }\right]$, and the initial speed was [0 m/s;0 m/s;0 m/s]. The UGV ran for a total of 108 s. It first accelerated for 5 s at $1{\;m/s}^{2}$ and then moved north for 10 s at a constant speed of 5 m/s. Next, it turned 90° to the east and continued to run at a constant speed for 20 s. After turning 90° to the south and running at a constant speed for 10 s, it continued to turn 90° to the east and ran at a constant speed for 20 s. Finally, after turning 90° to the north and decelerating at $-1{\;m/s}^{2}$ for 5 s, the UGV stopped. The true simulation trajectory is shown in Figure 3.

### 4.2. Sensor-Simulation-Parameter Setting

In this study, the gyroscope bias of the IMU in the INS mounted on the UGV was set to $\left[0.01^{\circ }/h;0.015^{\circ }/h;0.02^{\circ }/h\right]$, the accelerometer bias was [80 μg;90 μg;90 μg], the angular random-wander error was $0.001^{o}/\sqrt{h}$, the velocity random-wander error was $1\;\mu g/\sqrt{\mathrm{Hz}}$, and the sampling frequency was 100 Hz. The velocity-system error of the GNSS was 0.5 m/s, the position-system error was [1 m;1 m;1 m], and the sampling frequency was 1 Hz. The velocity-system error of the UWB was 0.5 m/s, the position-system error was [0.8 m;0.8 m;0.8 m], and the sampling frequency was 1Hz. The sensor parameters of the UGV were set as shown in Table 1.

### 4.3. Simulation Results and Analysis

**Figure 4:**
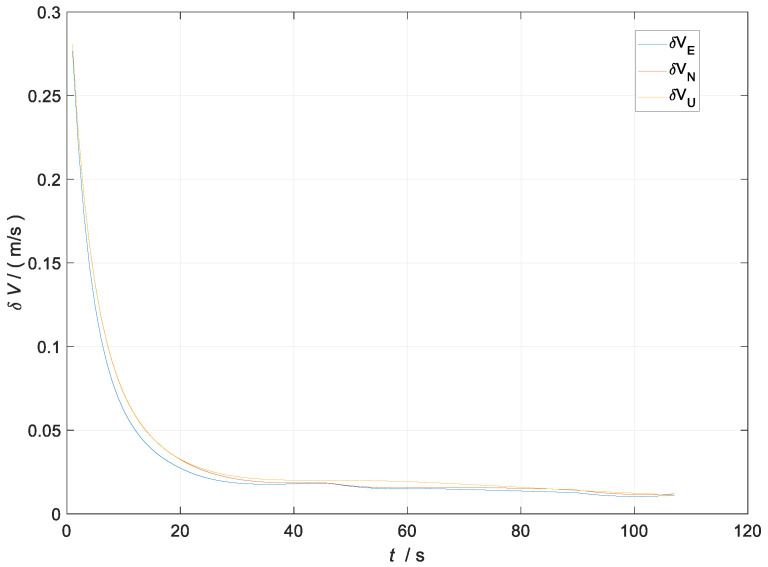
Diagram of the kinematic and static filtering velocity error of the ESKF based on INS/GNSS/UWB.

**Figure 5:**
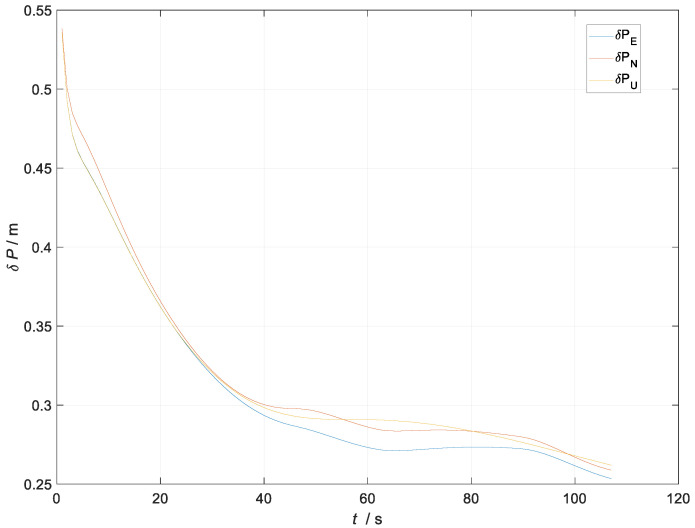
Diagram of the kinematic and static filtering position error of the ESKF based on INS/GNSS/UWB.

After designing the simulation trajectory related to the UGV, the measurement noise $v_{k}^{GNSS}$ of the kinematic filter was set to ${\left[0.5\;m/s;0.5\;m/s;0.5\;m/s;1\;m;1\;m;1\;m\right]}^{T}$ and the measurement noise $v_{k}^{UWB}$ of the static filter was set to ${\left[0.4\;m/s;0.4\;m/s;0.4\;m/s;0.8\;m;0.8\;m;0.8\;m\right]}^{T}$. The simulation experiments related to the kinematic and static filtering of the ESKF based on INS/GNSS/UWB were carried out, and the simulation results of the specific filtered velocity error and position error are shown in Figure 4 and Figure 5.

In Figure 4, the blue line represents the curve of the velocity error with time in the east direction during the filter process, the red line represents the curve of the velocity error with time in the north direction during the filter process, and the yellow line represents the curve of the velocity error with time in the sky direction during the filter process. All the units are in m/s.

In Figure 5, the blue line represents the curve of the eastward position error with time during the filter process, the red line represents the curve of the northward position error with time during the filter process, and the yellow line represents the curve of the skyward position error with time during the filter process. All of these measurements are in m.

The velocity error of the kinematic and static filtering of the ESKF based on the INS/GNSS/UWB converged around 20 s to 0.012 m/s. The position error converged around 40 s to 0.26 m. There was jitter in the position error at 50 s and 90 s; the reason for the jitter was the change in the position coordinates due to the turning maneuver of the carrier. The analysis above demonstrated that the kinematic and static filtering of the INS/GNSS/UWB-based ESKF can converge quickly.

**Figure 6:**
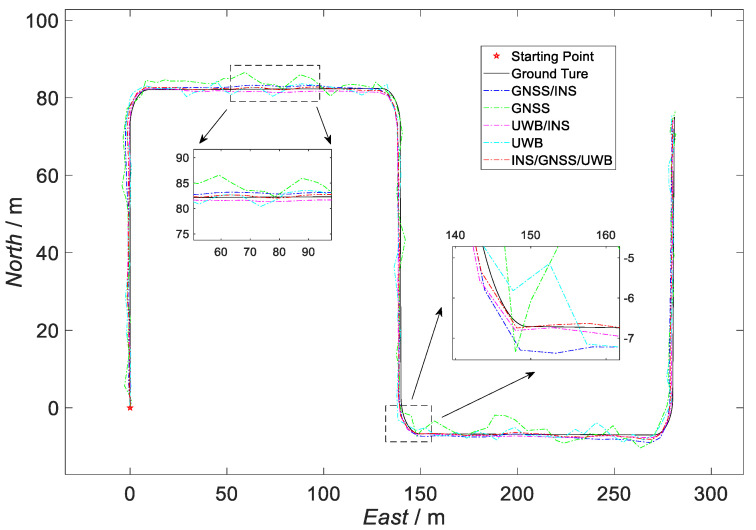
Comparison of the trajectories of the three schemes.

On this basis, this paper sets up two schemes for comparison between the loosely coupled GNSS/INS and the loosely coupled UWB/INS. The sensor parameters of the two schemes were consistent with Table 1; the observation error of the loosely coupled GNSS/INS was ${\left[0.5\;m/s;0.5\;m/s;0.5\;m/s;1\;m;1\;m;1\;m\right]}^{T}$, and the observation error of loosely coupled was UWB/INS ${\left[0.4\;m/s;0.4\;m/s;0.4\;m/s;0.8\;m;0.8\;m;0.8\;m\right]}^{T}$. The simulation-trajectory-comparison results obtained following the simulation-comparison experiments are shown in Figure 6.

The starting point in Figure 6 is the starting point of the UGV trajectory. In the figure, the black trajectory represents the real simulation trajectory, the blue dotted line represents the loosely coupled GNSS/INS simulation trajectory, the green dotted line represents the GNSS simulation trajectory, the purple dotted line represents the loosely coupled UWB/INS simulation trajectory, the blue dashed line represents the UWB simulation trajectory, and the red dashed line represents the trajectory of the kinematic and static filter simulation proposed in this paper.

According to Figure 6, in the straight-line operation phase, the simulation trajectory of the filter proposed in this paper was closer to the real trajectory than the simulation trajectory of the loosely coupled GNSS/INS scheme and the simulation trajectory of the loosely coupled UWB/INS scheme, as shown in the enlarged area on the left in the example diagram. The simulated trajectory of the method proposed in this paper also had a constraining effect on the error in the simulated trajectory of the two compared solutions during the turning-maneuver phase of the carrier. An example is given in the enlarged area on the right in the figure.

**Figure 7:**
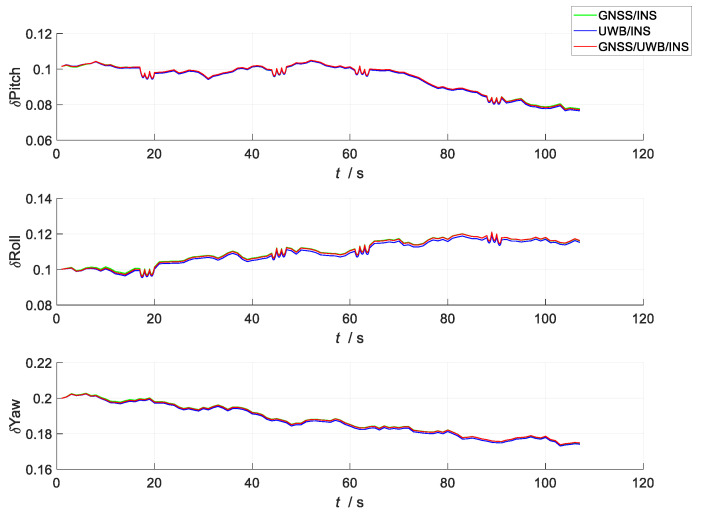
Comparison of attitude errors between the three schemes.

**Figure 8:**
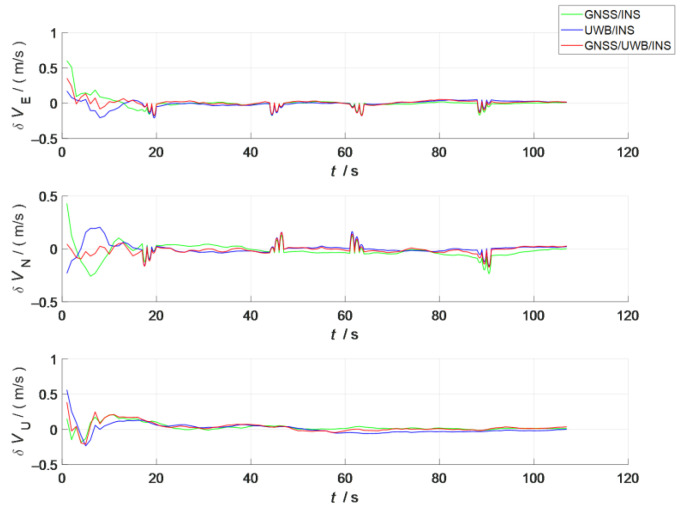
Comparison of velocity errors between the three schemes.

**Figure 9:**
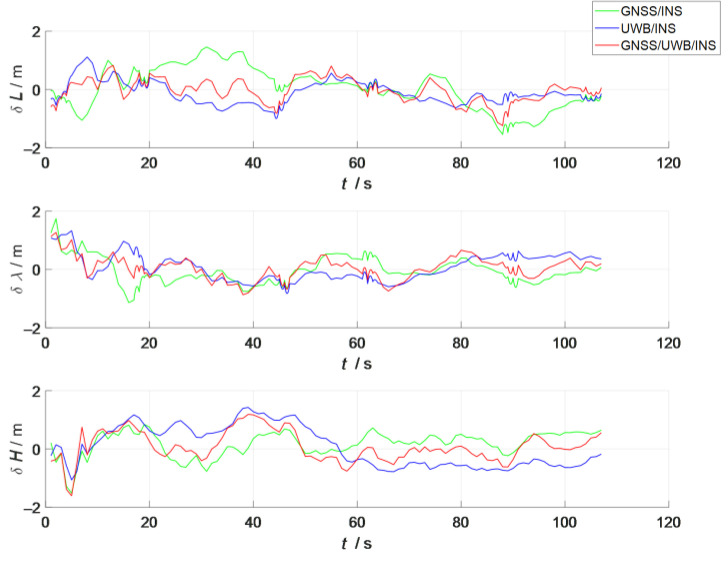
Comparison of position errors between the three schemes.

A comparison of the errors in the attitude, velocity, and position in the three simulation scenes i shown in Figure 7, Figure 8 and Figure 9. In the figures, the green line represents the errors in the attitude, velocity, and position versus time in the loosely coupled scheme GNSS/INS; the blue line represents the errors in the attitude, velocity, and position versus time in the loosely coupled UWB/INS scheme. The red line represents the errors in the attitude, velocity, and position versus time in the kinematic and static filtering of the ESKF based on the INS/GNSS/UWB proposed in this paper.

According to Figure 7, there were no significant differences between the errors in the yaw, roll, and pitch between the three schemes; the errors in the yaw and pitch angle both decreased with time, while the errors in the roll angle gradually increased with time, mainly because of the lack of feedback correction in the INS attitude data from both the kinematic ESKF and the static ESKF. Furthermore, the error curves for the yaw and roll angle both jittered around 20, 45, 60, and 90 s, which was mainly due to the carriers performing turning maneuvers at these times.

According to Figure 8, compared with the other two schemes, the absolute values of the velocity errors in the east–east, north, and sky directions of the schemes proposed in this paper were significantly reduced during the acceleration and steady phases from 0 to 20 s. After 20 s, the absolute values of the velocity errors all decreased slightly. The main reason for this was that all three schemes converged at around 20 s in the filter. Before this convergence, the difference between the three velocity errors was large because of the unstable filter. After the convergence, as the filter gradually stabilized and there was no coarse difference interference, the difference in velocity error between the three schemes was smaller.

According to Figure 9, the absolute values of the position errors in the longitude (east), latitude (north), and ellipsoidal altitude (down) of the proposed scheme in this paper were smaller than those in the other two schemes. However, since the fusion filter proposed in this paper fused the two ESKFs in a sequential form, the trend of its position-error-variation curve with time was similar to that of the kinematic ESKF (the ESKF based on the GNSS/INS). Therefore, in the construction of the overall kinematic and static filters based on the ESKF, the selection of the sensors in the kinematic ESKF largely affected the navigation accuracy of the overall filter. Therefore, the sensors in the kinematic ESKF should be selected to be more accurate and robust to correct the INS measurement data, thus improving the accuracy and robustness of the overall filter. Comparisons of the root mean square error (RMSE) and mean absolute error (MAE) of the attitude, position, and velocity of the three scenes are shown in Table 2 and Table 3.

According to Table 3, the overall RMSE of the attitude in the proposed kinematic and static filter of the ESKF based on the INS/GNSS/UWB was closer to that of the loosely coupled GNSS/INS and the loosely coupled UWB/INS; the overall RMSE of the velocity was reduced by 24.12% compared to the loosely coupled GNSS/INS and by 8.62% compared to the loosely coupled UWB/INS; the overall RMSE of the position was reduced by 19.84% compared to the loosely coupled GNSS/INS and by 17.44% compared to the loosely coupled UWB/INS. The overall RMSE of the position was reduced by 19.84% compared to the loosely coupled GNSS/INS and by 17.44% compared to the loosely coupled UWB/INS.

According to Table 4, compared to the loosely coupled GNSS/INS, the overall MAE of the velocity was reduced by 15.99%, and the overall MAE of the position was reduced by 23.70% in the kinematic and static filtering of the ESKF based on the INS/GNSS/UWB proposed in this paper. Compared with the loosely coupled UWB/INS, the overall MAE of the velocity was reduced by 12.46%, and the overall MAE of the position was reduced by 27.33%. The simulation results demonstrated that the method proposed in this paper can reduce the accumulation of errors caused by the integration of the INS and effectively improve the accuracy of the multi-source fusion navigation system based on kinematic and static filtering.

## 5. Comparative Experiments and Analysis

To verify the generalizability and robustness of the kinematic and static filtering of the ESKF based on the INS/GNSS/UWB algorithms proposed in this paper, two comparative experiments were set up. Experiment 1 analyzed the impact of the different sensors’ accuracies on the algorithms by setting the relevant parameters of the GNSS, UWB, and IMU. Experiment 2 compared the traditional ESKF-based federated filtering, ESKF-based dynamic and static filtering, and ESKF-based dynamic and static filtering with the switching strategy by setting up a simulation scene with reduced positioning accuracy under conditions of GNSS rejection and UWB non-sight-range conditions and by increasing the positioning errors of the GNSS and UWB in this environment.

### 5.1. Experimental Analysis of Sensor-Accuracy Comparisons

To analyze the impact of different sensor accuracies on the performance of the proposed algorithm, four different simulation scenes were set up, and the sensor parameters were set as shown in Table 4. In Scene 1, the UGV was equipped with a high-accuracy IMU, a high-accuracy GNSS (RTK-corrected GNSS), and a low-accuracy UWB. The measurement noise of the kinematic filter was set to ${\left[0.1\;m/s;0.1\;m/s;0.1\;m/s;0.2\;m;0.2\;m;0.2\;m\right]}^{T}$ and the measurement noise of the static filter was set to ${\left[0.4\;m/s;0.4\;m/s;0.4\;m/s;0.8\;m;0.8\;m;0.8\;m\right]}^{T}$. In Scene 2, the UGV was equipped with a high-accuracy IMU, a low-accuracy GNSS, and a high-accuracy UWB. The measurement noise of the kinematic filter was set to ${\left[0.5\;m/s;0.5\;m/s;0.5\;m/s;1\;m;1\;m;1\;m\right]}^{T}$ and the measurement noise of the static filter was set to ${\left[0.1\;m/s;0.1\;m/s;0.1\;m/s;0.2\;m;0.2\;m;0.2\;m\right]}^{T}$. In Scene 3, the UGV was equipped with a high-accuracy IMU, a low-accuracy GNSS, and a high-accuracy UWB. The measurement noise of the kinematic filter was set to ${\left[0.1\;m/s;0.1\;m/s;0.1\;m/s;0.2\;m;0.2\;m;0.2\;m\right]}^{T}$ and the measurement noise of the static filter was set to ${\left[0.1\;m/s;0.1\;m/s;0.1\;m/s;0.2\;m;0.2\;m;0.2\;m\right]}^{T}$. In Scene 4, the UGV was equipped with a low-accuracy IMU, a low-accuracy GNSS, and a low-accuracy UWB. The measurement noise of the kinematic filter was set to ${\left[0.5\;m/s;0.5\;m/s;0.5\;m/s;1\;m;1\;m;1\;m\right]}^{T}$ and the measurement noise of the static filter was set to ${\left[0.4\;m/s;0.4\;m/s;0.4\;m/s;0.8\;m;0.8\;m;0.8\;m\right]}^{T}$.

**Figure 10:**
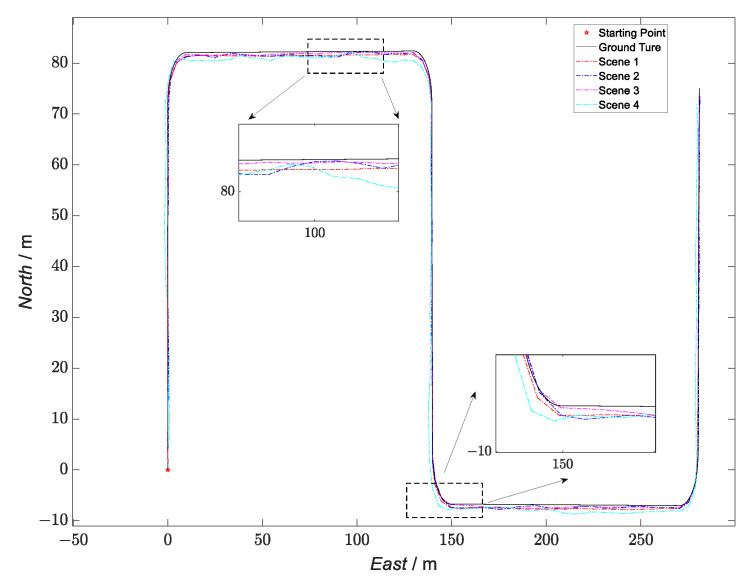
Comparison of the trajectories of the four scenes.

According to Figure 10, the kinematic and static filtering of the ESKF based on INS/GNSS/UWB proposed in this paper had good localization results in all four scenes, with different sensor accuracies. Because of the IMU, GNSS, and UWB on board the UGV in Scene 4 had the lowest accuracy, the UGV had a poorer localization effect in this scene than in the other three scenes. In the zoomed-in area of the straight-line running, the simulated trajectory of Scene 2 was closer to the real trajectory than those of Scene 1 and Scene 3, but the trajectory was not as smooth as the trajectory of the other two scenes. In the zoomed-in area for the turn run, the trajectory of Scene 3 was closer to the real value than the other three scenes, and the trajectories of Scene 1 and Scene 3 were relatively smooth compared to the other two scenes. The RMSE and MAE values for the four scenes are shown in Table 5 and Table 6.

According to Table 5 and Table 6, a comparison of the RMSE and MAE for Scene 1 and Scene 2 shows that the overall reduction in the attitude error for Scene 2 compared to Scene 1 was 9.19%, the overall reduction in the velocity RMSE for Scene 1 compared to Scene 2 was 40%, and the overall reduction in the position RMSE was 34.72%. The overall reduction in the pose error for Scene 2 was 8.94% compared to the overall reduction in the pose RMS error for Scene 1, the overall reduction in the velocity RMS error for Scene 1 compared to Scene 2 was 25%, and the overall reduction in the position RMS error was 32.76%. It can be deduced that compared to static filtering, the sensor accuracy of the dynamic filtering had a greater impact on the overall filtering accuracy. Because the algorithm proposed in this paper was used for sequential filtering, in the filtering design, the sensor with higher accuracy should enter into the overall filtering process first, as dynamic filtering.

**Figure 11:**
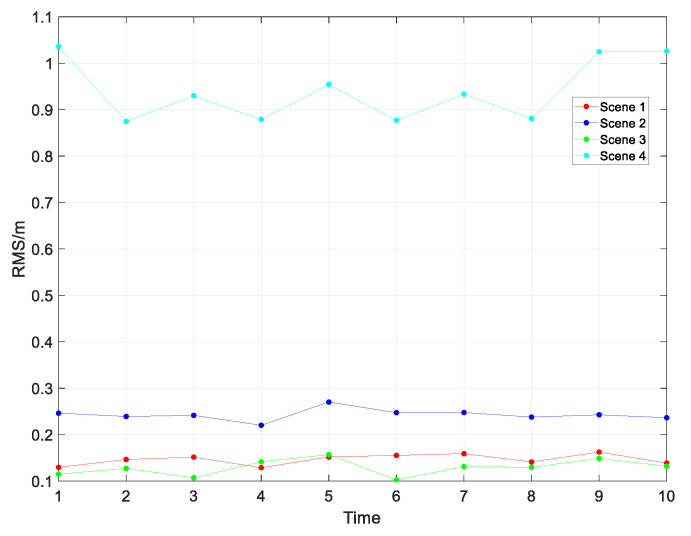
Experimental diagrams of Monte Carlo simulation of four scenes.

Based on the analysis of the errors in Scene 3 in Table 5 and Table 6, we learned that in the dynamic and static filtering based on the ESKF, the higher-accuracy GNSS (RKT corrected GNSS) and UWB had a better effect on the correction of the velocity and position of the low-accuracy IMU, and the overall positioning accuracy of the filter was higher than the positioning accuracy of the GNSS and UWB alone. However, as only the velocity and position information of the GNSS and UWB were used to correct the IMU during the observation of the dynamic and static filtering, which made its attitude errors accumulate gradually, the next step in the process was to equip the UGV with a dual-antenna GNSS, so that the GNSS could measure the heading (yaw) and course angle of the UGV, and then correct the attitude information of the IMU through the attitude angle obtained from the GNSS measurement. According to the analysis of the errors in Scene 4, we learned that the UGV can still show a relatively good positioning effect when equipped with a low-precision IMU, GNSS, and UWB. Based on this observation, 10 Monte Carlo experiments were conducted in the four scenarios, and the experimental results are shown in Figure 11.

### 5.2. Analysis of Complex-Environment-Simulation Experiments

To verify the robustness of the algorithm proposed in this paper in complex environments, we selected the relevant parameters and measurement noise of the sensors in Scene 3 in Section 5.1 as the experimental conditions and set up two environments with errors and three filtering-solution schemes. In Environment 1, from 10 s to 40 s, there was a non-line-of-sight situation for the UWB and occlusion between the tag and the base station, leading to a reduction in its positioning accuracy, which developed as follows. In the simulation scenario, the systematic error of the UWB was 20 times the initial error (taking 20 times as an example), the velocity’s systematic error was ${R^{\prime }}_{V_{UWB}}=20R_{V_{UWB}}=2\;m/s$, and the position’s systematic error was ${R^{\prime }}_{P_{UWB}}=20R_{P_{UWB}}=4\;m$. In Environment 2, from 66 s to 108 s, the GNSS was rejected and the receiver was located in the indoor scenario, resulting in a systematic error of the GNSS that was 20 times the initial error; the velocity’s systematic error was ${R^{\prime }}_{V_{GNSS}}=20R_{V_{GNSS}}=2\;m/s$ and the position’s systematic error was ${R^{\prime }}_{P_{GNSS}}=20R_{P_{GNSS}}=4\;m$. The GNSS and UWB positioning systems were normal at all the other times.

**Figure 12:**
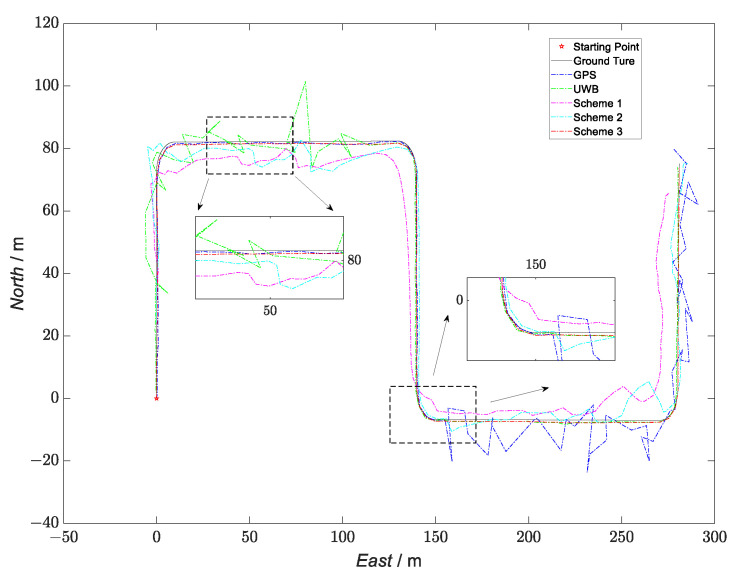
Comparison of the trajectories of the four schemes.

**Figure 13:**
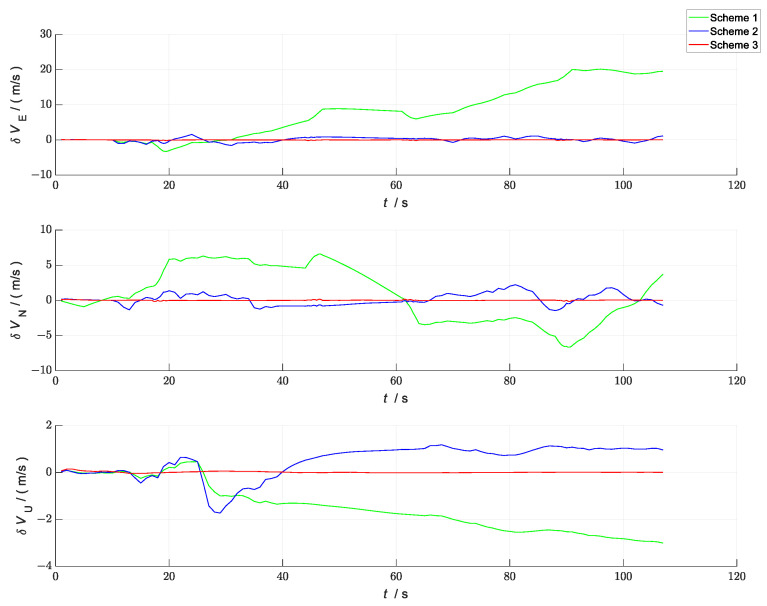
Comparison of the velocity errors between the three schemes.

**Figure 14:**
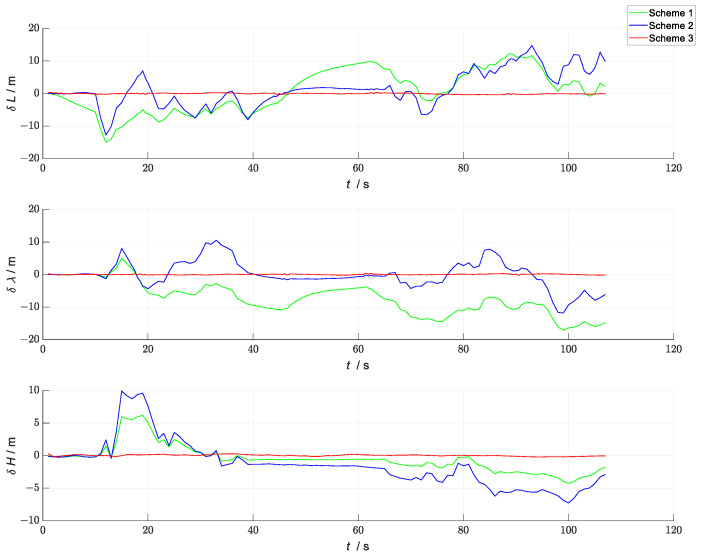
Comparison of the position errors between the three schemes.

In the setup of the filtering solution, ESKF-based federated filtering was used in Scheme 1. In the federated filtering, the two local filters were composed of INS/GNSS and INS/UWB, and the ESKF was used for the local filtering. To avoid matrix singularities in the inversion process due to the small values of the state vector and its covariance matrix in the ESKF, the local sensors were fused in the main filter in a feedback-free form, and the assignment factors of each local filter were $\beta_{i}=\frac{1}{2}(i=1,2)$. The kinematic and static filtering of the ESKF based on the INS/GNSS/UWB proposed in this paper was used in Scheme 2. Scheme 3, based on Scheme 2, introduced an evaluation system and switching strategy to block a particular sensor during the overall filtering in case its accuracy degraded. Taking this experimental scheme as an example, in the period of 10~40 s, because of the presence of large errors in the UWB data, the UWB data were shielded; only kinematic filtering was performed, and the kinematic filtering solution was output as the navigation solution for the overall filtering. In the period of 66~108 s, because of the large errors in the GNSS data, the GNSS data were shielded, and the overall filtering was only updated in time in the kinematic filtering. The UWB data were used to measure and update in the static filtering, the error-state vector was fed back to the INS, and the feedback INS data were obtained, which were then output as the navigation solution of the overall filtering. A comparison of the solved trajectories for the three schemes is shown in Figure 12, and the velocity-error and position-error comparisons are shown in Figure 13 and Figure 14, respectively.

According to Figure 13 and Figure 14, the kinematic and static filtering of the ESKF based on INS/GNSS/UWB with the evaluation system and switching strategy had smaller localization errors for velocity and position than Scheme 1 and Scheme 2. The error variation was smoother, mainly because of the shielding of the contaminated GNSS and UWB, which in turn improved the overall localization accuracy of the filtering. Compared to the federated filtering using the ESKF, the proposed algorithm had a higher overall positioning accuracy and a smoother error-variation curve. The RMSE and MAE of the three schemes are shown in Table 7 and Table 8, respectively.

According to Table 7 and Table 8, Scheme 3 had the highest accuracy, as the dynamic and static filtering had good plug-and-play capability, which shielded the contaminated sensors more quickly and conveniently, thus improving the overall system accuracy. Comparing Scheme 1 with Scheme 2, the RMSE and the MAE of the kinematic and static filtering of the ESKF based on the INS/GNSS/UWB in terms of attitude, velocity, and position were smaller than those of the ESKF-based federated filtering. Therefore, the proposed method had good robustness.

## 6. Conclusions

In this paper, we proposed a fusion-navigation algorithm with ESKF kinematic and static filtering of based on INS/GNSS/UWB. The algorithm not only corrected the velocity and position errors of the INS in turn by using the GNSS and UWB, but also ensured that the state and kinematic vectors of the overall system were small, which improved the convergence speed of the filter and reduced the errors in the linearization process. The simulation and control experiments demonstrated that the method proposed in this paper demonstrated faster filter convergence and improved the positioning accuracy by 21.98% and 13.03% compared to the loosely coupled GNSS/INS and UWB/INS methods, respectively. According to the comparison of the error-accumulation curves over time for the three navigation methods, the main performance of the overall system was largely influenced by the accuracy and robustness of the sensors in the kinematic ESKF because the kinematic and static filtering based on the ESKF was a sequential form of fusion filter. Therefore, in the construction of the integral kinematic and static filtering process based on the ESKF, the INS should be corrected first with a more accurate and robust sensor in the kinematic ESKF, in order to improve the performance of the overall filter system. In addition, through the simulation of the sensor parameters and complex-environment-simulation experiments, the proposed method was found to be applicable to sensors with different accuracies, with good generalization, plug-and-play, and robustness. In the next study, we will focus on improving the overall filtering-observation equation by equipping the UGV with multiple GNSS receivers so that the INS can be corrected using the GNSS heading (yaw) and course-angle information. We will also utilize additional sensors, such as visual odometers, binocular cameras, Bluetooth, etc., to optimize the data collected by the sensors through deep-learning algorithms, to improve the accuracy and robustness of the overall filtering.

## Figures and Tables

**Table 1:** Sensor parameter settings.

Sensor Type	Parameter		Value
IMU	Gyro error	bias	$\left[0.01{\;}^{\circ }/h;0.015{\;}^{\circ }/h;0.02{\;}^{\circ }/h\right]$
		random walk	$0.001{\;}^{o}/\sqrt{h}$
	Accelerometer error	bias	[80 μg; 90 μg; 90 μg]
		random walk	$1\;\mu g/\sqrt{\mathrm{Hz}}$
	Frequency		100 Hz
GNSS	Location		[1 m;1 m;1 m]
	Speed		0.5 m/s
	Frequency		1 Hz
UWB	Location		[0.8 m;0.8 m;0.8 m]
	Speed		0.4 m/s
	Frequency		1 Hz

**Table 2:** Comparison of the root mean square error (RMSE) of the three schemes.

RMSE	Pitch (″)	Yaw (″)	Roll (′)	VX (m/s)	VY (m/s)	VZ (m/s)	X (m)	Y (m)	Z (m)
Loosely coupled GNSS/INS	5.72	6.63	0.19	0.08	0.07	0.06	0.71	0.54	0.45
Loosely coupled UWB/INS	5.70	6.56	0.19	0.05	0.05	0.07	0.41	0.56	0.70
Kinematic and static filtering of ESKF based on INS/GNSS/UWB	5.73	6.62	0.19	0.05	0.04	0.07	0.40	0.46	0.51

**Table 3:** Comparison of the mean absolute error (MAE) of the three schemes.

MAE	Pitch (″)	Yaw (″)	Roll (′)	VX (m/s)	VY (m/s)	VZ (m/s)	X (m)	Y (m)	Z (m)
Loosely coupled GNSS/INS	5.70	6.62	0.19	0.04	0.05	0.04	0.59	0.42	0.38
Loosely coupled UWB/INS	5.68	6.55	0.19	0.04	0.03	0.05	0.34	0.47	0.63
Kinematic and static filtering of ESKF based on INS/GNSS/UWB	5.71	6.60	0.19	0.03	0.03	0.05	0.32	0.36	0.38

**Table 4:** Sensor-parameter settings for four different scenes.

Sensor Type	Parameter		Value			
			Scene 1	Scene 2	Scene 3	Scene 4
IMU	Gyro error	bias	$0.02{\;}^{\circ }/h$	$0.02{\;}^{\circ }/h$	$0.2{\;}^{\circ }/h$	$0.2{\;}^{\circ }/h$
		random walk	$0.001{\;}^{o}/\sqrt{h}$	$0.001{\;}^{o}/\sqrt{h}$	$0.08{\;}^{o}/\sqrt{h}$	$0.08{\;}^{o}/\sqrt{h}$
	Accelerometer error	bias	90 μg	90 μg	100 μg	100 μg
		random walk	$1\;\mu g/\sqrt{\mathrm{Hz}}$	$1\;\mu g/\sqrt{\mathrm{Hz}}$	$20\;\mu g/\sqrt{\mathrm{Hz}}$	$20\;\mu g/\sqrt{\mathrm{Hz}}$
	Frequency		100 Hz	100 Hz	100 Hz	100 Hz
GNSS	Location		0.2 m	1 m	0.2 m	1 m
	Speed		0.1 m/s	0.5 m/s	0.1 m/s	0.5 m/s
	Frequency		1 Hz	1 Hz	1 Hz	1 Hz
UWB	Location		0.8 m	0.2 m	0.2 m	0.8 m
	Speed		0.4 m/s	0.1 m/s	0.1 m/s	0.4 m/s
	Frequency		1 Hz	1 Hz	1 Hz	1 Hz

**Table 5:** Comparison of the root mean square error (RMSE) of the four scenes.

RMSE	Pitch (″)	Yaw (″)	Roll (′)	VX (m/s)	VY (m/s)	VZ (m/s)	X (m)	Y (m)	Z (m)
Scene 1	4.62	7.60	0.18	0.03	0.03	0.03	0.12	0.16	0.19
Scene 2	4.71	6.37	0.18	0.04	0.05	0.06	0.26	0.23	0.23
Scene 3	13.25	36.12	0.36	0.04	0.04	0.02	0.17	0.15	0.11
Scene 4	12.35	30.93	0.50	0.11	0.14	0.10	1.09	0.83	0.73

**Table 6:** Comparison of the mean absolute error (MAE) of the four scenes.

MAE	Pitch (″)	Yaw (″)	Roll (′)	VX (m/s)	VY (m/s)	VZ (m/s)	X (m)	Y (m)	Z (m)
Scene 1	4.59	7.53	0.18	0.02	0.02	0.02	0.10	0.13	0.16
Scene 2	4.69	6.33	0.18	0.02	0.03	0.03	0.21	0.19	0.18
Scene 3	10.29	34.86	0.27	0.02	0.02	0.01	0.13	0.12	0.09
Scene 4	9.89	24.18	0.42	0.09	0.10	0.07	0.95	0.66	0.54

**Table 7:** Comparison of the root mean square error (RMSE) of the three schemes in complex-environment-simulation experiments.

RMSE	Pitch (″)	Yaw (″)	Roll (′)	VX (m/s)	VY (m/s)	VZ (m/s)	X (m)	Y (m)	Z (m)
Scheme 1	833.72	740.69	221.99	11.46	3.91	1.23	5.49	11.86	1.72
Scheme 2	16.31	34.98	0.65	0.62	0.76	0.48	3.73	3.46	2.66
Scheme 3	30.36	38.64	0.86	0.04	0.04	0.02	0.18	0.25	0.13

**Table 8:** Comparison of the mean absolute error (MAE) of the three schemes in complex-environment-simulation experiments.

MAE	Pitch (″)	Yaw (″)	Roll (′)	VX (m/s)	VY (m/s)	VZ (m/s)	X (m)	Y (m)	Z (m)
Scheme 1	516.93	449.10	180.65	8.88	3.39	1.03	4.34	9.89	1.14
Scheme 2	13.54	33.02	0.56	0.46	0.56	0.27	2.44	2.37	1.56
Scheme 3	25.29	30.57	0.71	0.02	0.02	0.01	0.14	0.18	0.07

## Data Availability

Not Applicable.
